# Case of Mesenteric Cyst.
1Read at the Bristol Medico-Chirurgical Society, November 9th, 1904.


**Published:** 1904-12

**Authors:** A. W. Prichard

**Affiliations:** Senior Surgeon to the Bristol Royal Infirmary


### CASE OF MESENTERIC CYST.[1]

BY

A. W. PRICHARD,

*Senior Surgeon to the Bristol Royal Infirmary.*

---

THE subject of mesenteric cyst is one of considerable interest to all embryologists and abdominal surgeons, and the specimen that was removed in this case was one of those rare forms that have a cyst wall more than suggestive of the wall of part of the alimentary canal.

At present the belief is[2] that excepting hydatids and cysts

[1] Read at the Bristol Medico-Chirurgical Society, November 9th, 1904.

[2] Baumann, *Lancet*, 1904, i. 1272; Dowd, *Ann. Surg.*, 1900, xxxii. 515; J. Fawcett, *Tr. Path. Soc. Lond.*, 1902, liii. 406.

in malignant growths all mesenteric cysts are embryonic in origin. Rokitansky as far back as 1842 recognised the existence of such cysts, and believed them to be dilatations of lymph spaces. Dowd points out that included between the layers of the mesentery are found remnants of the Müllerian duct, of the Wolffian bodies, and of the vitelline duct, and suggests that the appearance of mesenteric cysts lies in the development of these cysts from such remnants. Their contents are a matter of chance, and according to what they contain they are named serous, chylous, sanguineous, or lymphatic. There is, however, one other form which has a wall resembling in structure the wall of the alimentary tube. These cysts probably owe their existence to their being originally diverticula or sequestrations from the intestine early shut off. Such is the case in the instance of the patient whose history I am now bringing forward.

C. B., æt. 15, was admitted at the Infirmary on August 31st, 1904. The history dates from when he was an infant in arms, since when he has always been liable to wind colic. His general health was fair, but often he had to get a certificate from his doctor to enable him to stay at home from school. The attacks of abdominal pain and swelling have been occurring irregularly about every two months, sometimes with and sometimes without diarrhœa. For the last ten days he has been kept in bed and has had six violent attacks with diarrhœa, five or six motions a day. He states graphically, "The lump comes up with pain that makes me groan; the lump goes, and then the pain passes off." The boy is pallid but not unhealthy looking. He has a small hare lip and does not want it touched. Heart and lungs natural. Abdomen looks quite natural till a contraction comes on, then a swelling about the size and shape of a stomach appears with peristalsis, but situated below the navel. It comes suddenly, is firm and not tender. Tickling the parietes will produce it. The parietes are pushed forward by it. It is resonant, and after staying some minutes or so it disappears with a gurgle. Urine 1022, faint trace of albumen. He was watched for a few days, and had two longer attacks than usual. Sometimes the swelling came without the pain. No diagnosis was made as to the cause; he was believed to be suffering from some unusual form of chronic intestinal obstruction. Operation on October 5th. I made a three-inch incision below the umbilicus; a huge mass was found pushing forward the bowel. The portion of bowel most pushed forward was, I believe, the transverse colon. It looked like a huge intussusception or a hernia of the stomach between the layers of the mesocolon. In fact, when I had incised the peritoneum over it, it was so like a stomach that I prolonged my skin incision to prove that the stomach was in its normal place. I opened the cyst and about 15 oz. of fluid was let out. I then proceeded to free it from its attachments, stripping off the peritoneum with considerable difficulty and hemorrhage. A pedicle was found at the vertebral attachment and tied off, and after all bleeding points had been attended to the abdomen was closed without drainage. Transfusion was performed during the operation, and on removal to his bed the boy was in a very collapsed condition. The cyst looked smooth on its outer surface, and was filled with glairy, dark blood-stained fluid. The inner surface was rough and papillated, and felt soft and sticky like a mucous membrane. Except for an attack of indigestion on the sixth day from drinking milk he made an uninterrupted recovery, and is now well and has had none of the old pain. An interesting point in his convalescence was that his pulse was never under 90 even when he was kept quite still. If he moved about his pulse was 120–130. He was not breathless or weak.

### PATHOLOGICAL REPORT ON SECTIONS.

By J. J. S. LUCAS, M.B. Lond.

Sections were cut from two different parts of the specimen, including the whole of the cyst wall. They were exactly similar in appearance. They resemble very closely the duodenum in structure. Internally are long glands lined with cylindrical epithelium and containing mucous cells. Their blind ends are racemose in character, and spread out into the muscularis mucosæ like Brunner's duodenal glands. Outside this are two muscular coats separated by loose areolar tissue. The inner coat is cut obliquely and probably represents a circular coat, while the outer is cut transversely and is continuous over the whole section and represents a longitudinal coat. The conclusion is that the tumour is of the structure of the small intestine.

